# Pharmacy Internal Controls: A Call for Greater Vigilance during the COVID-19 Pandemic

**DOI:** 10.3390/pharmacy8040216

**Published:** 2020-11-15

**Authors:** Andrew N. Mason

**Affiliations:** School of Medicine, Juntendo University, Bunkyo 113-8421, Japan; m.andrew.at@juntendo.ac.jp

**Keywords:** COVID-19 pandemic, internal controls, pharmacy inventory, drug diversion, fraud

## Abstract

For businesses that store physical goods, managing product inventories and financial cost accounting controls are critical. Pharmacies are under considerable scrutiny, due to the nature of their merchandise, making internal controls even more vital. Due to the emergence of COVID-19 and government mitigation strategies, the US economy has seen significant macro- and microeconomic effects. COVID-19 has changed the pharmacy working environment, which could theoretically increase rates of employee drug diversion. Therefore, better inventory management could reduce the misuse of pharmaceutical drugs from fraudulent and drug diversion activities. The author explored secondary findings to create a multidisciplinary conceptual analysis of the reasons why internal controls executed with greater diligence may be needed to avoid damaging financial, legal, and health outcomes. The author also provides a review of available internal control methods that can be used to mitigate diversion.

## 1. Introduction

Pharmacy businesses operate as specialized merchandise organizations, providing healthcare products to the marketplace. To meet consumer demands in a timely fashion, pharmacies must store adequate types and quantities of products in inventory. According to the World Health Organization (WHO), managing pharmaceutical product inventory effectively is vital to the success of a pharmacy organizational unit [1]. To manage inventory, it is important for pharmacies to develop comprehensive internal controls. Internal controls are defined as the methods used within an organization to safeguard assets, enhance reliability of accounting controls, increase efficiency of operations, and ensure compliance with laws and regulations [2].

Pharmacy operations primarily consist of the sale of medical supplies and prescription/nonprescription medications. It is the dispensing of medications that make pharmacy inventory unique. Non-prescription medicines, also called over-the-counter (OTC) medication, are typically on floor room shelves accessible by customers, while prescription medications are kept behind the counter and only reachable by employees. Prescription medications can be further broken down into controlled substances and non-controlled substances. Controlled substances are often medications that are stimulants (e.g., methylphenidate) or analgesics for pain (e.g., oxycodone), which have a higher risk for abuse. Non-controlled medications include those for the treatment of common chronic conditions (e.g., blood pressure, diabetes, etc.) that have less, or no, potential for euphoria inducing effects.

Typically, inventory represents the highest value of current assets within pharmacy operations. In recent years, the value of inventory has grown significantly as has the number of different pharmaceutical products available [3,4]. As active drug components in medications degrade, over time, and have a finite shelf life, controls must be used to ensure medications are dispensed before they lose efficacy and go to waste. Spoilage of medications can result in lost profits, or negative health outcomes for a patient if dispensed. Moreover, pharmacy inventory is highly mobile, and therefore, easy to divert. This is compounded by the fact that some medications have a higher risk for substance abuse (i.e., controlled substances). As such, the industry is highly regulated by multiple regulating bodies (Food and Drug Administration, Drug Enforcement Administration, State Board of Pharmacy, etc.) that can impose stiff penalties for professional and accounting errors. For these reasons, it has always been imperative that pharmacies develop and operationalize sound internal controls.

Generally, pharmacies have been able to develop effective internal controls and meet set standards. However, it is important to revisit standard business practices and perhaps implement new operating procedures in the wake of current events that significantly impact business operations and staff. Sometime in 2019, a novel coronavirus, COVID-19 emerged. COVID-19 spread rapidly and threatened the health and economies of the world’s nations. On 11 March 2020, the World Health Organization (WHO) declared COVID-19 a global pandemic. COVID-19 and the United States (U.S.) government interventions (e.g., nationwide lockdowns) designed, to stem the spread of the virus, have both caused significant macro- and microeconomic effects. This research highlights the possible need for better inventory management to reduce the misuse of pharmaceutical drugs from fraudulent and drug diversion activities. More specifically, findings from the literature show the growing risk for fraud and diversion in the pharmacy workplace. 

## 2. Methods

The research model consisted of a search of secondary research. The findings were synthesized by a narrative review. More specifically, secondary research was conducted by searching two bibliographic healthcare databases. Publications from influential medical journals were searched for from Scopus and Pubmed. The search of relevant databases was conducted by searching recent scholarly journal publications (from 2000 to present) using a combination of specific keywords to gather pharmacy controls data. The restriction of articles within the last 20 years was placed to keep the articles somewhat contemporary and to gather enough articles for review to draw meaningful conclusions. Pharmacy controls keywords included: Pharmacy inventory management, pharmacy internal controls, drug fraud, and drug diversion. Table 1 displays the number of search hits from each keyword searched within the specified 20-year time frame.

## 3. Literature Review Summary

Sommersguter-Reichmann et al. summarized the staggering costs of healthcare fraud across multiple countries by pointing out that fraud is estimated to account for 3% to 8% of a given nation’s health expenses [5]. Furthermore, they reported as much as 10% of public health expenditures in Germany were due to fraud, and their research estimated that US Centers for Medicare and Medicaid Services loses approximately 100 billion dollars annually. 

The US Congress enacted the Drug Supply Chain Security Act (DQSA) in 2013, which gave powers to the US Food and Drug Administration (FDA) to employ a digital tracing system to follow designated prescription drugs within the US drug supply chain [6]. This act enhanced the capability of the FDA to protect consumers from access to diverted drugs, as well as contaminated drugs. In addition, the act established national licensure standards for distributors, which are annually monitored by the FDA. 

Despite the regulatory authorities’ attempts to stem drug fraud and diversion, success has been elusive. For example, a recent US government report found several shortcomings by the Drug Enforcement Administration or DEA [7]. The report stated that the DEA has been slow in responding to the significant diversion of opioids since 2000. Furthermore, the report found the DEA to be ineffective at detecting diversion with existing data systems and administrative enforcement tools. In addition, the report found that existing regulations and policies do not adequately require accountability from registrants, nor are they effective at preventing diversion of pharmaceutical opioids. Lastly, the report called on the DEA to more effectively address the drug diversion crisis.

With the high volume of drug fraud and diversion occurring, it is little wonder that Beninger argues for greater pharmacovigilance diligence and calls for greater government oversight to monitor regulation gaps that exist from the manufacturing of pharmaceutical drugs to when they are administered and ultimately consumed by patients [8]. Furthermore, Moniveena et al. call for the regulatory community to introduce improved pharmaceutical drug disposal processes to reduce drug diversion [9]. 

The outset of the COVID-19 pandemic is making matters even worse, as vital pharmaceutical drug supply chains have been disrupted. For example, Newton and Bond found that many medical supplies were repurposed to use in the fight against COVID-19, resulting in the potential for substandard and/or falsified products [10]. Furthermore, Newton and Bond argue that the impact of COVID-19 pandemic on supply chain disruptions has increased substandard and falsified medical products on a global scale [10]. 

In addition, several studies have found that patients face increased critical threats from counterfeit and fraudulent medicines that exist within the pharmaceutical drug supply chain [11,12,13,14]. Fraudulent and counterfeit medicines are fake medicines that are marketed as authorized medicines. These products can be dangerous to patients and consumers because they often contain ingredients that are incorrect or sub-standard, and may have incorrect doses [15]. Recent increases in fraudulent medicines have produced treatment failures, harmful side effects, and even deaths [16,17].

Existing literature supports the need for pharmacy settings to establish and operationalize effective inventory controls. Religioni et al. evaluated the usefulness of effective inventory controls when they investigated several Polish hospital pharmacies [18]. Audits included direct supervision, secondary data analysis, and an independently designed questionnaire. The questionnaire assessed hospital drug distribution procedures, drug dispensing volumes, hazardous drugs preparation processes, and information about electronic information systems used in medication orders and dispensing processes. The findings demonstrated inventory control shortcomings. Fortunately, the study revealed that external audits can be very beneficial, especially when the pharmacy is introducing new regulations into the workplace.

## 4. Discussion of Fraud and Diversion

Fraud has always been an ever-present danger in the pharmacy setting. The most likely form of fraud in a pharmacy is the diversion of medication for personal use or financial gain. Kenna and Wood found that incidences of pharmacists diverting drugs for personal use is of significant concern [19]. This is not surprising, considering how accessible medications are to pharmacists. As seen in the Literature Review Summary, fraud and diversion are significant concerns that have been compounded by the current pandemic. Some of the ways the pandemic could impact fraud and diversion in the pharmacy setting are discussed below.

The COVID-19 pandemic and resultant mitigation strategies have led to many changes to the U.S. economy and society at large. Statewide lockdowns have been used to encourage social distancing and decreased business operations for all except workers who are deemed “essential.” The results of these governmental restrictions have caused a significant decrease in national GDP, as well as a massive increase in those filing for unemployment benefits [20]. Couple these financial woes with concerns over contracting COVID-19, along with a reduction in social interaction, and we have the potential for disastrous outcomes. Unsurprisingly, in this climate, the U.S. Center for Disease Control confirmed a significant uptick in mental health issues—namely an increase in anxiety and substance abuse among the general population. This uptick in mental health concerns was even more prevalent in “essential” workers [21]. Pharmacies have been deemed “essential,” resulting in continued operation throughout the pandemic. It is possible that the greater demands at work and a more difficult work environment are the cause for increased stress and mental strain in pharmacies [22]. Moreover, the use of personal protective equipment (PPE) may further exacerbate this situation. For example, Veluri asserts that patients can experience an increase in paranoia when interacting with others wearing face masks because masks interfere with the patient’s ability to detect a clinician’s empathy [23]. Therefore, patients are reluctant to volunteer personal information, making it difficult for clinicians to develop good patient rapport, which can interfere with the provision of effective treatment and can reduce efficiency. Pal et al. also found that wearing face masks creates barriers to viewing facial expressions, inhibiting efficient provider-patient communication [24]. This, in turn, could increase the time needed for pharmacist-patient interactions, resulting in increased stress and mental strain as pharmacists attempt to complete their other supervisory and administrative roles in the reduced time remaining. Taking the Fraud Triangle into account, this environment could influence the rates of diversion. The Fraud Triangle is defined as the three most likely reasons that someone might commit fraud: Opportunity, financial pressure, and rationalization [2].

The opportunity for theft of pharmacy products is high. Pharmacies are bustling environments with a lot of movement by staff: pharmacists, pharmacy technicians, and salesclerks. The pharmacy supervisors, usually pharmacists, have a number of responsibilities, making it difficult for them to keep tabs on what everyone is doing in the pharmacy at any given time. Furthermore, medications are small, lightweight, and stored in large quantities. As such, the removal of a few pharmaceuticals during business hours can go unnoticed until the total quantity of diverted goods reaches a certain threshold. The increased demands placed on pharmacists during the COVID-19 pandemic makes for a more mentally taxed pharmacist thereby interfering with their ability to provide oversight. This, in turn, makes for greater opportunities for drug diversion to occur. In addition, with pandemic induced anxiety and depression on the rise, pharmacists are filling higher prescription volumes which increases inventory turnover [25]. With higher inventory turnover, small inventory losses could become more difficult to notice [26].

For a pharmacy healthcare worker who is under financial pressure, diversion may seem like a quick solution. As stated above, pharmaceuticals are highly mobile, and many controlled medications are also highly liquid. The street value for opiates (analgesics) is currently about $1 per milligram [27]. Therefore, a 100-count bottle of oxycodone/acetaminophen 10/325 could theoretically be sold on the street for $1000, which is far more than the cost to acquire it through legal channels. The economic realities of COVID-19 lockdowns have likely resulted in a decrease in household income for some pharmacy staff members whose spouses or family members have lost wages due to their working in “nonessential” industries that have been shut down or curtailed from government pandemic mitigation polices. It would be reasonable that pharmacy staff members might feel pressure to consider drug diversion activities as a temporary solution to meet financial shortcomings.

Finally, rationalization may play a key role in medication diversion. Often diversion within a pharmacy setting results from the actions of pharmacy technicians who are estimated to make up three-fourths of all drug diversion cases [28]. There are no definitive studies explaining the reasons that pharmacy technicians constitute such a high percent of drug diversion cases. However, it is commonly understood that all pharmacy staff are being asked to maximize output in an increasingly difficult work environment as reported by Algunmeeyn et al [29]. Levy argues that heightened stress can lead pharmacy employees to rationalize that self-medicating can be justified as a coping strategy to overcome workplace challenges [30]. To complicate matters, the risk for rationalization of drug diversion activities may increase because of the stresses and financial pressures brought on from the COVID-19 pandemic. Regardless of the rationalized cause, it is important to control drug diversion because it has a significantly greater impact on society than the sum of the monetary value of the drugs involved [5].

## 5. Implications for Pharmacy Internal Controls

A solution to reducing fraud is the proper execution of internal controls, consisting of all methods within an organization to safeguard assets, enhance reliability of accounting records, increase efficiency of operations, and enhance compliance with laws and regulations [2]. To realize effective internal control of drugs, pharmacies in both the hospital and community settings must implement control policies and procedures into their operations. Accounting controls help pharmaceutical businesses manage their product inventory levels, as well as monitor the financial costs associated with their product inventories. With respect to product inventory control, pharmacies typically utilize personal visual inspections and/or computerized methods. Visual verification of product levels can be accomplished by physical inspections taken on a random basis or taken at periodic intervals (e.g., daily, weekly, monthly, yearly). In either case, with the visual inventory accountability method, human spot checks are conducted to determine if the products on hand match with the listing of products that should be present. When product levels are found to fall below desired inventory levels, purchase orders are issued to replenish needed product inventory. 

While, visual inventory controls are useful, the most common method of inventory control is the perpetual method, whereby, computerized systems are used to monitor product inventory levels on a continuous basis [3,31]. More specifically, with perpetual inventory controls, hand-held scanning devices are used to read product identification information that is digitally encoded on barcode labels, which are located on the product’s packaging. Products are scanned as they go into, and out of, inventory, allowing product inventory levels to be updated in real time. Perpetual inventory technology and developments in logistics have allowed most pharmacies to convert to just-in-time inventory methods to keep inventory levels low and improve inventory turnover. In just-in-time inventory systems, pharmacies order inventory that are depleted and receive it the following day, significantly reducing the risk for spoilage.

Pharmacies can also use a hybrid of visual and perpetual inventory control methods. That is, random or routine interval visual counts can be conducted in conjunction with computerized perpetual control systems. With the hybrid approach, pharmacists can compare product inventories that are known to be in stock via visual inspection to reports generated by computer related technologies. Any discrepancies that may be observed between the visual and computerized controls may indicate human counting error, software error, or possible fraudulent activity. 

Pharmacy inventory, by its nature, is subject to a higher risk of fraudulent activity. Therefore, many internal controls should be implemented in the pharmacy setting. To reduce fraudulent activities, pharmacy controls must include the following: segregation of duties, physical controls, independent internal verification, and human resource controls [2]. 

Employees’ duties must be segregated within the pharmacy to ensure double checks on inventory coming in as well as outgoing product. There is usually one pharmacy technician responsible for ordering and receiving medications into inventory. A pharmacist must then double check the physical count of the medications being added to inventory alongside the order’s sales invoice. This reduces the likelihood of a medication being ordered and then, subsequently, diverted before getting added to physical inventory.

Physical controls are a legal requirement in pharmacies [32]. While, over-the-counter medications are generally regarded as safe for the general populace and are freely available to customers on the floor, prescription medications are not. As such, prescription medications are kept behind the counter of a pharmacy, out of reach to the general populace. The only way to acquire these medications is with a legal prescription from a licensed physician. Substances designated as Control Level II (CII) (controlled medications categorized with the highest risk of abuse), are subjected to further physical controls and must be kept in a safe at all times. In a community pharmacy setting, pharmacists are usually the personnel with the key to this safe and must get involved any time a controlled substance is dispensed. In hospital settings, controlled substances should be loaded in automated dispensing machines (e.g., Omnicell, Mountain View, CA, USA), and in order to access the controlled substance, the user must have a login that has the authority to withdraw controlled substances. In both cases, withdrawals must be documented and time stamped, so that discrepancies can be traced back to the individuals involved.

As stated earlier, pharmacies usually do periodic inventory checks to reconcile differences in what is actually on hand with what the perpetual inventory system has recorded. In small independent pharmacies, pharmacy management, or the on-duty Pharmacist-in-Charge, is often responsible for verifying inventory records monthly and annually. Random checks must be performed when management is suspicious of fraudulent activity. Those that are involved in the check should not be involved in the day-to-day management of inventory, and thus, are useful as independent auditors. Auditors must also sign off on the results of the inventory check. Therefore, it is in their best interest to catch errors and alert the appropriate authorities (e.g., local law enforcement, DEA, etc.) if the discrepancies spotted cannot be appropriately resolved. Some larger pharmacy chains (e.g., Walgreens, Deerfield, IL, USA) have units within the organization which have responsibilities for tracking inventory usage and spotting fraudulent activity. Due to the high-risk nature of pharmacy inventory diversion, an extra layer of independent verification must exist. U.S. State Pharmacy Boards, for the respective U.S. states, send auditors for periodic and random site visits to make sure adequate inventory documentation practices including that the documentation is accurate.

Human resource controls are also necessary in a pharmacy setting. While, all personnel are potential candidates for diversion and abuse of medications, some are higher risk hires. To determine this risk, hiring managers often perform background checks on potential hires to determine if they have a history of criminal activity or drug-related charges. To reduce the likelihood of fraud in pharmacies, new hires could be subjected to drug tests to restrict the hiring to candidates who are not currently abusing drugs and have no prior history of drug-related charges. Furthermore, it is important that inventory control procedures be developed that balance the pharmacists’ need to provide vital healthcare services and the need to reduce fraud and drug diversion. That is, pharmacy employees provide front line retail services and, as such, have considerable strains placed upon their time. Therefore, the frequency of conducting random checks and visual counts should be determined at the micro level because these controls can interrupt normal operations. For instance, the frequency of conducting random checks or internal visual counts could be determined by metrics that take into consideration the number of employees and prescription volume associated with a given pharmacy’s operations. 

## 6. Conclusions

Due to the nature of their merchandise, pharmacies have unique and inherent risks for fraud and diversion. The COVID-19 pandemic and government interventions have resulted in a number of changes—both in society at large and on an individual level. These changes may impact the level of drug diversion taking place in the pharmacy setting. Executing effective internal controls with greater diligence during these tumultuous times will provide the best means for pharmacies to combat fraudulent activities. Pharmacists and pharmacies, as the gatekeepers for medications, have a social responsibility to the communities in which they operate, in order to keep potentially harmful and addictive medications out of the hands of those that do not have a clinical need for such products. Medications that find their way into the community at large through unscrupulous channels can have far reaching effects. Diverted pharmaceutical drugs are a problem facing pharmacies, and establishing effective internal controls can play an important role in reducing diversion and should perhaps receive increased attention during times of crises. The findings illustrate the need for additional research to quantify the impact the COVID-19 pandemic has had on diversion rates.

## Figures and Tables

**Table 1:** Literature Review Metrics (2000–2020).

Keywords Searched	Database Search Hits	
	Scopus	Pubmed
Pharmacy inventory management	10	4
Pharmacy internal controls	0	0
Drug diversion	747	447
Drug fraud	15	837
